# Multi-Tissue Transcriptomics Analysis of the Effects of Ammonia Nitrogen Stress on Metabolism, Immunity, and Comprehensive Stress Responses in *Megalobrama amblycephala*

**DOI:** 10.3390/ani16142139

**Published:** 2026-07-09

**Authors:** Mingguo Lu, Yang Guo, Silei Xia, Jinjuan Wan, Kun Wu, Hui Cao, Wuxiao Zhang, Aimin Wang

**Affiliations:** 1 College of Marine and Biology Engineering, Yancheng Institute of Technology, Yancheng 224051, China; 2024816083@stu.njau.edu.cn (M.L.); 2024816082@stu.njau.edu.cn (Y.G.); susanxia1990323@126.com (S.X.); 2 Key Laboratory of Agricultural Environmental Vicrobiology, Ministry of Agriculture and Rural Affairs, College of Life Sciences, Nanjing Agricultural University, Nanjing 210095, China; hcao@njau.edu.cn; 3 Freshwater Fisheries Research Institute of Jiangsu Province, Nanjing 210017, China; wanjinjuan.a@163.com; 4 College of Marine Sciences, South China Agricultural University, Guangzhou 510642, China; wk@scau.edu.cn

**Keywords:** *Megalobrama amblycephala*, ammonia-N stress, metabolism, immunity, transcriptome

## Abstract

In order to reveal the molecular response characteristics of different tissues of *Megalobrama amblycephala* under ammonia nitrogen stress, the liver, gill, muscle, kidney and brain tissues of juvenile *Megalobrama amblycephala* (12.05 ± 0.04 g) under 25 mg/L ammonium chloride stress were used as the research objects. The transcriptome sequencing technology was used to systematically analyze the transcriptional expression changes. A total of 204.42 Gb transcription data were obtained, and a total of 3039 DEGs were detected, of which 1331 genes were up-regulated and 1708 genes were down-regulated. Ten DEGs were randomly selected for quantitative qRT-PCR analysis, and the results confirmed that the transcriptome results were reliable. Multi-organ synergy analysis suggested that overlapping DEGs (e.g., *ZNF239*, *DMBT1*, *NLRC3*, *MHC-I*, and *CCL8*) may exhibit similar or opposing regulatory patterns across organs, potentially reflecting complementary and coordinated mechanisms in immune regulation, inflammatory responses, energy allocation, and detoxification strategies among organ systems. GO and KEGG pathway enrichment analysis of differentially expressed genes showed that ammonia nitrogen mainly affected immune inflammation-related processes, material transport and degradation, protein homeostasis maintenance, oxidative stress defense, membrane lipid metabolism remodeling, and energy metabolism regulation. In different tissues, genes related to specific functions are enriched according to their physiological roles: in the liver, genes related to detoxification and damage clearance are enriched; in the gill, genes related to barrier defense and antioxidant/detoxification are enriched; in the kidney, genes related to damage response and metabolic regulation are enriched; in the brain, genes related to membrane lipid homeostasis and protective stress are enriched; and in the muscle, genes related to energy metabolism and structural function adjustment are enriched. This study elucidated the multi-tissue response characteristics of *Megalobrama amblycephala* under ammonia nitrogen stress at the transcriptome level, providing fundamental data and references for further clarifying the mechanisms of ammonia nitrogen toxicity in fish and screening stress-responsive marker genes.

## Simple Summary

This study suggests involvement in the multi-tissue response characteristics of blunt snout bream under ammonia nitrogen stress at the transcriptome level. Enrichment analysis indicated the involvement of immune inflammation-related processes, material transport and degradation, protein homeostasis maintenance, oxidative stress defense, membrane lipid metabolism remodeling, and energy metabolism regulation. Different tissues show different molecular response characteristics according to their physiological functions: in the liver, genes related to detoxification and damage clearance were differentially expressed; in the gill, genes related to barrier defense and antioxidant/detoxification were differentially expressed; in the kidney, genes related to damage response and metabolic regulation were differentially expressed; in the brain, genes related to membrane lipid homeostasis and protective stress were differentially expressed; and in the muscle, genes related to energy metabolism and structural and functional adjustment were differentially expressed.

## 1. Introduction

Ammonia nitrogen is an important environmental stress factor in the process of aquaculture, and its increase in concentration will pose a serious threat to the growth, health, and productivity of aquatic organisms [1]. In the process of intensive aquaculture, the excessive use of feed and the excretion of animal manure can easily lead to the high concentration of ammonia nitrogen in water [2]. The biological toxicity of ammonia nitrogen in water is mainly due to the fat-soluble NH_3_, which can freely penetrate the biofilm, destroy the gill function, inhibit ammonia excretion, and lead to the double accumulation of ammonia in vivo and in vitro, causing an increase in blood ammonia concentration and the outbreak of reactive oxygen species (ROS) [3], changing the activity of antioxidant-related enzymes to induce oxidative stress and interfere with the balance of oxidation in vivo [4]. It induces the expression of inflammation-related genes and the maturation of pro-inflammatory cytokines in fish, activates an inflammatory response and causes cell damage, and it also affects the expression of genes related to metabolism and immune response [5,6].

*Megalobrama amblycephala* is a kind of fish in the genus *Megalobrama of Cyprinidae*, Cypriniformes, commonly known as Wuchang fish. It is highly sensitive to ammonia nitrogen and is prone to show typical stress responses, such as gill tissue damage, elevated serum cortisol, and disorder of antioxidant enzyme activity [7,8]. This sensitivity makes *Megalobrama amblycephala* an ideal model for studying the mechanism of ammonia nitrogen stress in aquatic organisms [7,9]. According to statistics, in 2023, the production of *Megalobrama amblycephala* in China will reach 738,700 tons (the seventh largest freshwater fish in China) [10]. It is one of the important economic edible fish in China, and its health is directly related to breeding efficiency and market supply. Therefore, in-depth study of the response of *Megalobrama amblycephala* under ammonia nitrogen stress is of great significance for improving the development of healthy aquaculture.

In recent years, with the development of high-throughput sequencing technology, transcriptomics has become one of the powerful tools to analyze the molecular mechanism of environmental stress response in aquatic organisms, which can systematically reveal the adaptation strategies of organisms to environmental changes [11,12,13]. For example, chronic ammonia stress has been shown to significantly inhibit the growth of *Megalobrama amblycephala*, and its mechanism involves the down-regulation of the growth hormone/insulin-like growth factor (GH/IGF) axis [14]. In addition, the growth inhibition mediated by the tuberous sclerosis complex 2 (*TSC2*) gene through the AMPK/mTOR signaling pathway was also found in *Megalobrama amblycephala* [15]. Ammonia nitrogen stress also affects the muscle quality of *Megalobrama amblycephala* and changes its energy metabolism and redox system [16]. However, previous studies have primarily focused on single tissues or specific pathways, limiting our understanding of coordinated multi-organ responses and potential systemic regulatory patterns under acute ammonia stress. A system-level analysis integrating multiple tissues is essential because each organ plays a distinct yet interconnected role in the organism’s response to ammonia stress. Gill is the core barrier of ammonia nitrogen absorption, excretion and osmotic regulation [9,17,18]; the liver is the hub of ammonia metabolism, detoxification and energy supply [19]; the kidney is an important organ for ammonia excretion, ion balance and osmotic regulation [20]; muscle is mainly responsible for ammonia storage, transport and energy metabolism [21,22]; and the brain is one of the sensitive targets of ammonia toxicity [5]. Isolated tissue analyses fail to capture the integrated physiological and molecular network that coordinates the whole-body stress adaptation. Therefore, a systematic analysis of the multi-organ oxidative stress linkage regulation network through spatiotemporal dynamic transcriptomics is necessary to elucidate potential multi-organ response patterns.

In light of this, we hypothesize that ammonia stress induces tissue-specific, yet coordinated, transcriptional responses associated with metabolism and immune regulation. This study aimed to systematically investigate the effects of ammonia stress on multiple key tissues (including liver, gill, brain, muscle, and kidney) of *Megalobrama amblycephala* through multi-tissue transcriptome analysis. We identified differentially expressed genes and specific pathways activated under ammonia stress and elucidated the potential molecular mechanisms involved in metabolic reprogramming, immune regulation, and integrated stress responses in *Megalobrama amblycephala*. These findings contribute to a more comprehensive molecular map of ammonia toxicity and offer potential targets for precise intervention against ammonia-induced toxicity.

## 2. Materials and Methods

### 2.1. Experimental Design

*Megalobrama amblycephala* used in this experiment were obtained from the fish farm at Freshwater Fisheries Research Center, Chinese Academy of Fishery Sciences (Wuxi, China). Prior to the ammonia nitrogen stress exposure, fish were acclimatized for two weeks in 200 L tanks containing 100 L of water. During this acclimatization period, fish were fed a commercial diet (Wuxi Tongwei Feedstuffs Co., Ltd., Wuxi, China) twice daily (8:00 and 17:00). Feeding was suspended 24 h before commencing the stress experiment.

### 2.2. Acute Stress Experiment

Based on our previous study on acute ammonia nitrogen stress in *Megalobrama amblycephala*, the 24 h LC50 for juvenile fish was 65.277 mg/L, and a concentration of 25 mg/L was found to induce significant physiological responses [23]. Therefore, this experiment established two ammonia nitrogen concentration groups: 0 mg/L (control group) and 25 mg/L, each with three independent tank replicates (*n* = 3), with 20 fish per tank (total *n* = 120, average body weight of 12.05 ± 0.04 g). All fish were healthy, similar in size, and randomly allocated into the six tanks. A high concentration of ammonium chloride solution was added to the culture barrel as an ammonia source to adjust to the required final concentration [24]. The actual total ammonia nitrogen concentrations in the control group and the experimental group were 0.052 ± 0.003 mg/L and 25.31 ± 0.09 mg/L, respectively, determined by the Nesslerization method [3]. The concentration of free ammonia in the control group and the experimental group was 0.006 ± 0.000 mg/L and 2.430 ± 0.008 mg/L, respectively [25]. Perform regular daily sewage discharge and systematic inspections during the stress period; the water tank is supplied by groundwater and provides supplementary oxygen. All tanks are supplied with dechlorinated tap water, and water bodies are replaced according to actual needs (one-third of the volume is replaced each time); the tanks were filled with dechlorinated and aerated water, maintaining the following conditions: temperature at 25.44 ± 0.11 °C, pH at 7.52 ± 0.10, dissolved oxygen (DO) at 9.28 ± 0.23 mg/L, and an artificial light regime of 12 h light (from 08:00 to 20:00) and 12 h dark (from 20:00 to 08:00) for the fish. Water temperature, pH, and DO were measured using a multiparameter water quality instrument (YSI Inc., Yellow Springs, OH, USA) [24,26].

### 2.3. Sample Collection

After 24 h of ammonia nitrogen stress, healthy juvenile fish were randomly selected from six breeding barrels using MS-222 (100 mg/L; Sigma Chemical Company, St. Louis, MO, USA) sampling on ice (liver, gill, kidney, brain, and muscle). According to the method of Wang et al. [12], tissues taken from 10 individuals per tank were mixed (i.e., there were 3 mixed samples used for transcriptome detection for each of the two groups, *n* = 3, with tank as the experimental unit). Therefore, 6 mixed samples were collected for each tissue type, resulting in a total of 30 samples from 5 tissues being subjected to transcriptome analysis. Finally, an additional 3 fish per tank were dissected to collect tissue samples for analysis of relevant gene expression levels (*n* = 3, with tank as the experimental unit). All the samples were rinsed with normal saline and placed in a 2 mL centrifuge tube for labeling (liver-LC/LN, gill-GC/GN, kidney-KC/KN, brain-BC/BN, and muscle-MC/MN; note: C is the control group, and N is the experimental group), immediately stored at −80 °C for subsequent RNA extraction.

### 2.4. RNA Quantification and Qualification

RNA concentration and purity were determined using the NanoDrop 2000 spectrophotometer (Thermo Fisher Scientific, Wilmington, DE, USA). RNA integrity was evaluated with the RNA Nano 6000 Assay Kit on the Agilent Bioanalyzer 2100 system (Agilent Technologies, Santa Clara, CA, USA).

### 2.5. Library Preparation for Transcriptome Sequencing

Total RNA (1 µg per sample) served as the input material for library preparation. Eukaryotic mRNA was enriched by magnetic beads with Oligo (dT); fragmentation buffer was added to randomly interrupt mRNA; the first cDNA strand was synthesized by random hexamers using mRNA as template, and then the second cDNA strand was synthesized by adding buffer, dNTPs, RNase H and DNA polymerase I. The cDNA was purified by AMPure XP beads (Beckman Coulter, Beverly, CA, USA). The purified double-stranded cDNA was subjected to terminal repair, an A-tail was added, and the sequencing adapter was connected, and then the fragment size was selected with AMPure XP beads. Finally, the cDNA library was obtained by PCR enrichment.

After the library construction was completed, Qubit2.0 was used for preliminary quantification, and Agilent 2100 was used to detect the insert size of the library until it was in line with expectations. Finally, the effective concentration of the library was accurately quantified by the Q-PCR method (the effective concentration of the library > 2 nM) to complete the library inspection. After the library inspection was qualified, different libraries were pooled according to the amount of target offline data and sequenced using the Nova seq6000 platform (Illumina, San Diego, CA, USA). The sequencing read length was PE150.

### 2.6. Gene Assembly and Annotation

Raw data (raw reads) in fastq format were first processed through in-house Perl scripts. In this step, clean data (clean reads) were obtained by removing reads containing adapter, reads containing poly-N, and low-quality reads from raw data. At the same time, Q20, Q30, GC-content and sequence duplication level of the clean data were calculated. All the downstream analyses were based on clean data with high quality. Trinity software (v2.4.0) was used to assemble the high-quality Clean Data sequence to generate the transcript sequence of each gene. The parameter settings were: --min_contig_length 300 --min_kmer_cov 3 --KMER_SIZE 27, and the longest transcript in each gene was taken as Unigene.

Unigene sequences were aligned against the NR, Swiss-Prot, GO, COG, KOG, and KEGG databases using BLAST (v2.2.26) [27]. Subsequently, HMMER software (v3.2.1) was employed to search the Pfam database to obtain functional annotation for the unigenes. BLAST preferences: blastall -p blastx -e 1 × 10^−5^.

### 2.7. Differential Expression Analysis

Bowtie2 (V4.4.7) was used to compare the sequencing reads with the unigene library. According to the comparison results, the expression level was estimated in combination with RSEM. Then, the Pearson correlation coefficient was calculated to detect the repeatability between samples. DEGs were performed on the transcriptome data of the ammonia nitrogen group and the control group. Calculate the difference multiple using the DESeq2 package in R (v3.6.2); threshold setting: Filter out the genes whose sum of read count values of the two groups is less than 2. Screening criteria: |log2 (FC)| ≥ 1 and FDR (Benjamini–Hochberg correction method was used to obtain the *p* value of significant difference) < 0.05. Finally, the DEGs were analyzed by GO, COG, KOG and KEGG enrichment analysis.

### 2.8. Real-Time Quantitative PCR (RT-qPCR) for Genetic Verification

In order to verify the accuracy of RNA-seq data, 10 genes were selected from DEGs of five tissues for RT-qPCR. Primers were designed using NCBI and Primer3-Plus based on the gene sequences obtained from RNA-seq (Table 1). Total RNA was extracted using the Solarbio Total RNA Extraction Kit (Solarbio, Beijing, China). The HiScript^®^ II One Step RT-qPCR SYBR Green Kit (Vazyme, Nanjing, China) was used for cDNA synthesis and amplification. Reagents were mixed in RNase-free centrifuge tubes according to the manufacturer’s protocol. The amplification efficiencies for all genes were comparable, ranging from 90% to 110%. β-Actin was used as the internal reference gene to normalize expression levels. Reaction specificity was verified by dissociation curve analysis. Gene expression levels were calculated using the relative quantitative 2^−ΔΔCt^ method. Statistical analysis was conducted using IBM SPSS Statistics 27, and graphical representations were generated using GraphPad Prism 9.5.

## 3. Results

### 3.1. Sequencing Data and Quality

High-throughput transcriptome sequencing of 30 samples (Table 2) generated 204.42 Gb of transcriptome data. All samples exhibited Q30 base percentages ≥ 92.85% and GC contents ranging from 45.8% to 49.2%, confirming high sequencing quality and providing a solid basis for subsequent analyses [12,29].

A total of 849,712 Transcripts and 476,067 unigenes were assembled (Table 3). The N50 lengths for transcripts and unigenes were 1812 bp and 842 bp, respectively, and the assembly integrity was high. Unigene length distribution analysis revealed: 95,981 (20.16%) unigenes spanning 300–500 bp, 69,570 (14.61%) spanning 500–1000 bp, 29,856 (6.27%) spanning 1000–2000 bp, and 280,660 (58.95%) exceeding 2000 bp. All raw sequencing data have been deposited in the NCBI Sequence Read Archive under accession number PRJNA1337661.

### 3.2. Gene Function Annotation

In order to obtain the complete functional information of the gene, the unigene sequence was compared with the seven functional databases of NR, Swiss-Prot, GO, COG, KOG, KEGG and Pfam databases, and a total of 118,320 unigene annotation information was obtained (Table 4).

**Figure 1:**
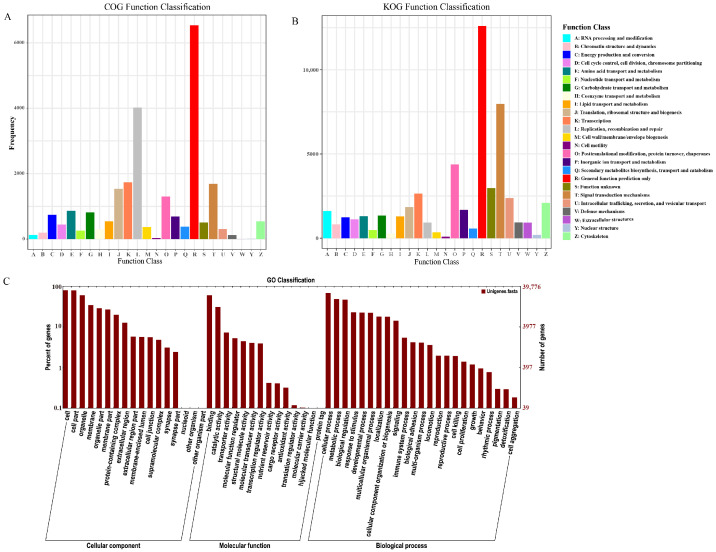
COG, KOG, and GO annotation classification statistical diagram of expressed genes ((**A**) stands for COG annotation classification statistical chart, (**B**) stands for KOG annotation classification statistical chart, and (**C**) stands for GO annotation classification statistical chart).

**Figure 2:**
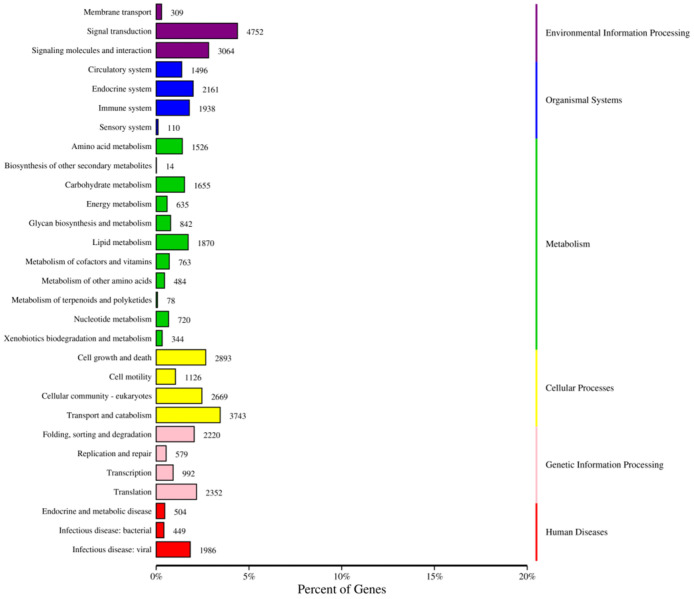
KEGG annotation classification statistical diagram of expressed genes.

The COG database annotated 18,695 genes into 25 categories, with general function prediction only and replication, recombination, and repair being the most frequently represented categories (Figure 1A). The KOG database annotated 46,341 genes into 25 categories, among which general function prediction only, signal transduction mechanisms, and post-translational modification, protein turnover, and chaperones were the top three most abundant (Figure 1B). The GO database annotated a total of 39,776 genes, categorizing them into three major domains: Molecular Function, Cellular Component, and Biological Process. Within the Cellular Component domain, genes were predominantly associated with cell, cell part, and organelle. In the Molecular Function domain, genes were primarily concentrated in binding, catalytic activity, and transporter activity. For the Biological Process domain, genes were mainly involved in metabolic process, cellular process, and biological regulation (Figure 1C). The KEGG database annotated 108,414 genes, and the assembled genes were mainly assigned to functional categories such as signal transduction, transport and catabolism, signaling molecules and interaction, cell growth and death, translation, folding, sorting and degradation, lipid metabolism, carbohydrate metabolism, amino acid metabolism, and immune system (Figure 2).

### 3.3. Annotation and Enrichment Analysis of DEGs

**Figure 3:**
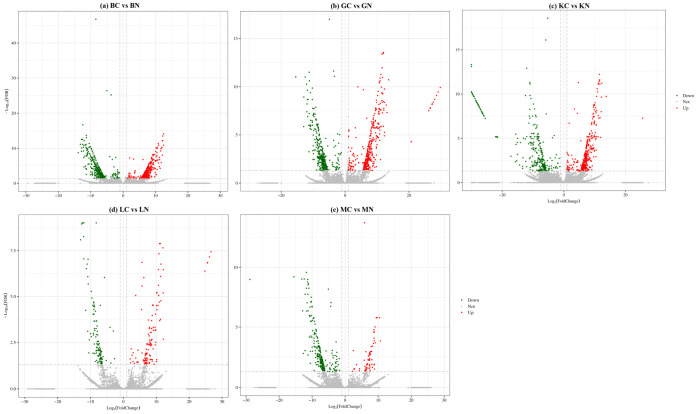
Compared with the control group, the differential gene expression of five tissues in the ammonia nitrogen group was compared (the red point indicates that the gene expression was up-regulated, the green point indicates that the gene expression was down-regulated, and the gray point indicates that the gene expression was not changed).

**Figure 4:**
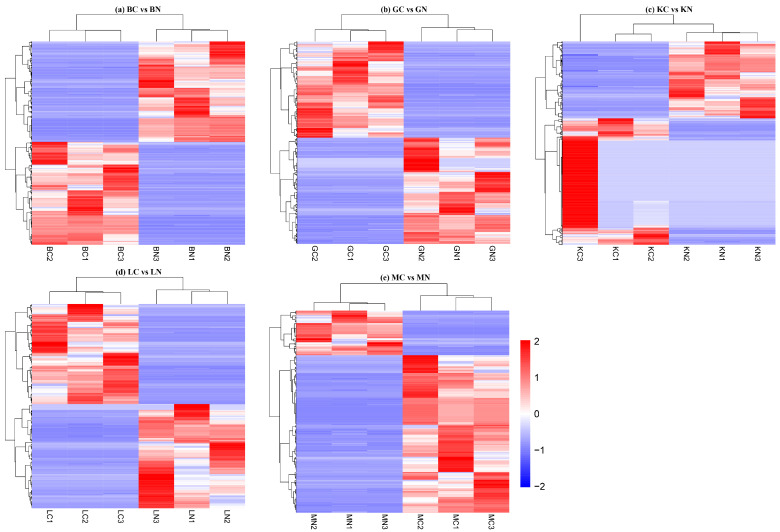
Cluster diagram of DEGs.

**Figure 5:**
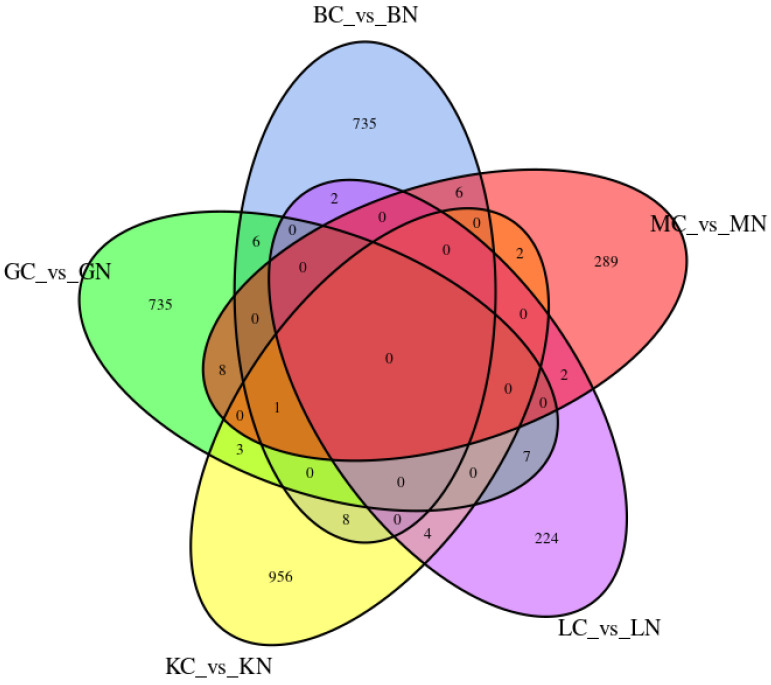
Venn diagram of DEGs.

Transcriptome analysis of the five tissues identified a total of 3039 DEGs (Table 5, Figure 3), comprising 1331 up-regulated and 1708 down-regulated genes. Gill tissue exhibited the highest number of DEGs, while liver tissue showed the lowest (Figure 3). Cluster analysis of DEGs in each tissue between the control and ammonia nitrogen groups revealed significant differences in DEGs expression across tissues at both concentrations (Figure 4). Comparison of DEGs among the five tissues identified overlapping genes between tissues (Table 6, Figure 5).

### 3.4. Functional Annotation and Enrichment Analysis of DEGs

#### 3.4.1. COG and KOG Functional Annotation Analysis of DEGs

**Figure 6:**
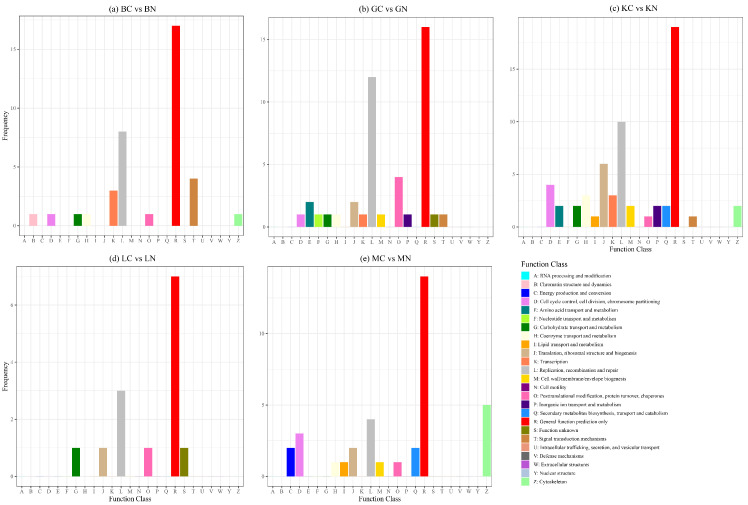
COG annotation classification statistics of DEGs in five organ samples.

**Figure 7:**
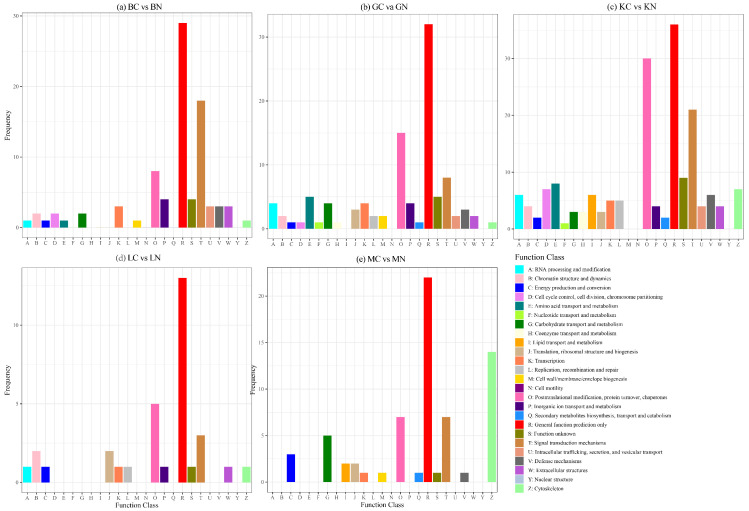
KOG annotation classification statistics of DEGs in five organ samples.

Among the DEGs screened from five tissue samples, 162 and 380 were annotated to the COG and KOG databases, respectively (Table 7). The annotation of the COG database mainly focused on general function prediction, DNA replication and repair, signal transduction, protein processing, and cell structure maintenance. Among them, the kidney and gill have the widest functional response range, and the brain and muscle show obvious structural and signal regulation characteristics, while the liver is more reflected in the relatively concentrated stress repair response (Figure 6). The KOG database is mainly enriched in general function prediction, post-translational modification/protein turnover, signal transduction, ion transport, cytoskeleton, and metabolism-related functions. Among them, the kidney is the most complex, followed by the gill, and the muscle has obvious characteristics in terms of cytoskeleton. The brain is more reflective of changes in nerve signals and ion homeostasis, and the liver is relatively based on basic regulation and homeostasis maintenance (Figure 7).

#### 3.4.2. GO Functional Annotation Analysis of DEGs

**Figure 8:**
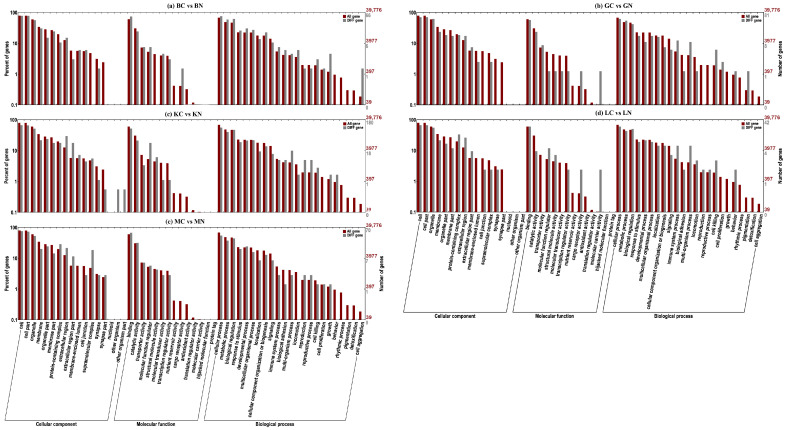
GO annotation classification statistics of DEGs in five organ samples.

A total of 439 DEGs identified across the five tissue samples were annotated using the GO database (Table 7). The GO database categorizes gene functions into three branches: Biological Process (BP), Molecular Function (MF), and Cellular Component (CC). The analysis found that, in addition to some common enrichment pathways, in the Biological Process, the enrichment of brain and gill in the metabolic process was greater than that of the other three tissues. The enrichment degree of gill and liver in the immune system process was higher than that in the other three tissues. In Molecular Function, the number of differentially expressed genes in liver and gill was higher in molecular carrier activity (Figure 8).

#### 3.4.3. KEGG Functional Annotation and Enrichment Pathway Analysis of DEGs

**Figure 9:**
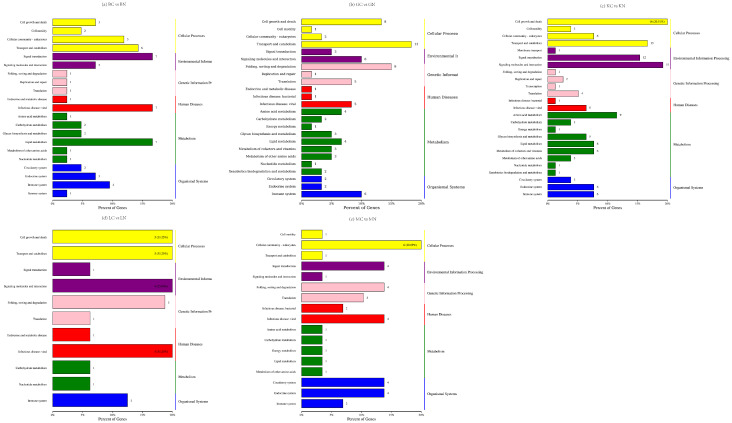
KEGG annotation classification statistics of DEGs in five organ samples.

A total of 478 differentially expressed genes screened from five tissue samples were annotated to the KEGG database (Table 7). The enrichment degree and type of each tissue in the KEGG database are different. The DEGs in the liver are mainly annotated in functional categories such as cell growth and death, transport and catabolism, signaling molecules and interaction, and folding, sorting and degradation (Figure 9d); the DEGs in gill tissue were mainly annotated in functional categories such as transport and catabolism, folding, sorting and degradation, cell growth and death, immune system and signaling molecules and interaction, and involved a certain proportion of amino acid metabolism, lipid metabolism and exogenous metabolism-related categories (Figure 9b); the DEGs in muscle tissue were mainly distributed in cellular community-eukaryotes, signal transduction, folding, sorting and degradation, circulatory system and endocrine system, accompanied by a certain degree of translation and immune-related functional changes (Figure 9e); the DEGs in the kidneys were mainly concentrated in cell growth and death, signaling molecules and interaction, signal transduction, transport and catabolism and amino acid metabolism, and involved in lipid metabolism, cofactor and vitamin metabolism, immune system and endocrine system (Figure 9c). The DEGs in brain tissue were mainly distributed in functional categories such as signal transduction, lipid metabolism, transport and catabolism, cellular community-eukaryotes, immune system, and endocrine system (Figure 9a).

**Figure 10:**
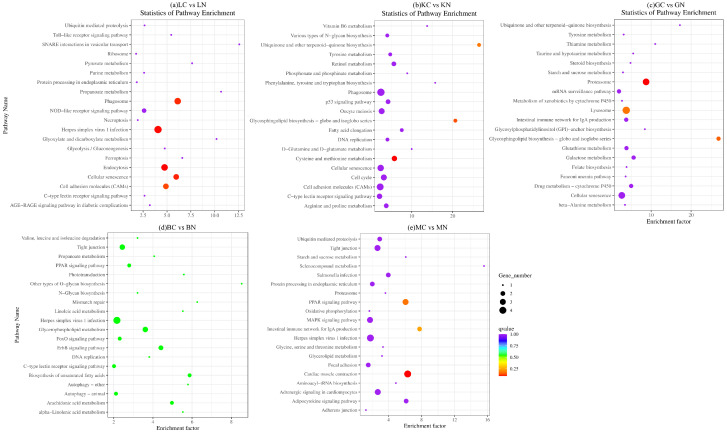
KEGG pathway enrichment scatter diagram of DEGs ((**a**) is liver tissue samples, (**b**) is kidney tissue samples, (**c**) is gill tissue samples, (**d**) is brain tissue samples, and (**e**) is muscle samples).

Further KEGG pathway enrichment analysis of each tissue showed that different tissues showed obvious tissue-specific response characteristics after ammonium chloride stress. The enrichment pathway mainly involved immune-inflammatory response, material transport and degradation, energy metabolism remodeling, oxidative stress, cell fate regulation, and other biological processes. The DEGs in the liver were mainly enriched in phagosome, endocytosis, NOD-like receptor signaling pathway, ferroptosis, cellular senescence and other pathways, among which the phagosome and endocytosis pathways were significantly enriched (Figure 10a); the DEGs in kidney tissue were mainly enriched in Phagosome, p53 signaling pathway, cell cycle, cellular senescence, cysteine and methionine metabolism, and Arginine and proline metabolism (Figure 10b); the DEGs in gill tissues were mainly enriched in proteasome, lysosome, glutathione metabolism, drug metabolism–cytochrome P450, metabolism of xenobiotics by cytochrome P450, taurine and hypotaurine metabolism and cell senescence-related pathways (Figure 10c); the DEGs in brain tissue were mainly enriched in tight junction, glycerophospholipid metabolism, biosynthesis of unsaturated fatty acids, arachidonic acid metabolism, alpha-linolenic acid metabolism, FoxO signaling pathway and autophagy pathways. Compared with other tissues, the significance of brain tissue enrichment pathway is relatively weak, but it can still reflect the change trend of membrane lipid metabolism, barrier function and stress protection of nerve tissue under ammonium chloride stress (Figure 10d); the DEGs in muscle tissue were mainly enriched in cardiac muscle contraction, oxidative phosphorylation, PPAR signaling pathway, MAPK signaling pathway, ubiquitin mediated proteolysis, proteasome, glycerolipid metabolism and adipocytokine signaling pathway (Figure 10e).

### 3.5. Validation of Transcriptome Results by RT-qPCR

**Figure 11:**
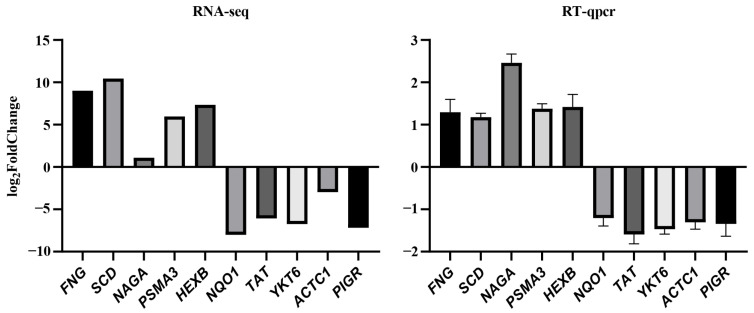
The RNA−seq results were verified by RT−qpcr, and 10 genes were randomly selected and normalized with β−action as reference genes (2^−ΔΔCt^ relative quantitative method).

Ten DEGs were randomly selected for RT-qPCR validation of the RNA-seq results, with β-actin serving as the internal reference gene for normalization. As shown in Figure 11, although the expression levels varied among the genes, their expression trends were consistent with the RNA-seq data, confirming the accuracy of the transcriptome analysis.

## 4. Discussion

A total of 476,067 unigenes were assembled in this transcriptome, but only 118,320 unigenes were annotated, and the annotation coverage was low. This result is very common in the study of non-model organisms or complex genomes, which may be caused by the inherent limitations of the reference database. In this study, comparison between the ammonia nitrogen-stressed group and the control group revealed organ-specific variation in the number of DEGs. The gill tissue, being the primary site of direct ammonia nitrogen exposure and exchange [3,30], exhibited the highest number of DEGs. This indicates that gills might play a crucial role in sensing and initiating the response to ammonia nitrogen stress. Ammonia nitrogen stress facilitates significant influx of exogenous NH_3_ through the gills and skin, leading to tissue accumulation and consequent damage to the fish immune system [31]. The number of overlapping DEGs between tissues is limited, and the expression direction is not completely consistent, indicating that the molecular response of blunt snout bream to ammonia nitrogen stress is mainly tissue-specific changes, and there are a small number of cross-tissue co-expression characteristics. These genes can be used as the basis for subsequent functional verification and key candidate gene screening, but the related functions still need to be further verified.

The annotation results of the COG and KOG databases mainly focus on stress signal perception, protein homeostasis maintenance, DNA repair, ion transport, and cell structure regulation. However, KOG can better reflect the complex response of blunt snout bream to ammonia nitrogen than COG, especially in protein processing, cytoskeleton, ion metabolism, and defense response. From the perspective of tissue differences, the functional classification of DEGs in kidney and gill tissues is the most abundant, suggesting that they are most sensitive to ammonium chloride stress. Kidney tissue plays an important role in ammonia excretion, homeostasis maintenance, and injury repair [32]. The gill tissue is mainly characterized by enhanced protein quality control, signal transduction, and metabolic regulation, reflecting its barrier response characteristics as the first contact interface of external environmental stimulation [33]. DEGs in brain tissue are more involved in signal transduction and ion metabolism, suggesting that ammonium chloride stress may affect the homeostasis of the nervous system. The cytoskeleton-related categories in muscle tissue are more prominent, indicating that it may have obvious structural remodeling [34,35]. The functional changes in liver differential genes are relatively concentrated, mainly involving general functional regulation, protein homeostasis maintenance, and stress repair processes. In general, the results of COG and KOG annotations confirm each other, indicating that ammonium chloride stress can cause systematic and tissue-specific molecular responses in multiple tissues of blunt snout bream in signal transduction, protein homeostasis, ion regulation, damage repair, and cell structure maintenance.

GO enrichment analysis showed that brain and gills were highly enriched in the “metabolic process” category, indicating that these tissues had a strong metabolic response to ammonia nitrogen stress to maintain their functional homeostasis and detoxification. Gill tissue, directly exposed to high ambient ammonia concentrations that threaten normal cellular physiology [36,37], responds by up- or down-regulating key metabolic pathways [38,39,40]. The nervous system plays an important role in coping with ammonia nitrogen stress [41,42]. Given the neurotoxic potential of high ammonia levels, which can cause neurological dysfunction and mortality [43], the brain likely modulates its metabolic activity to protect neurons and sustain normal neurotransmission [42,43]. Gills and liver may play a central role in the immune defense of aquatic animals. This is supported by findings in *Oreochromis niloticus*, where hypoxia-induced gill immunosuppression may parallel effects of ammonia stress [38]. Similarly, studies on *Scylla paramamosain* demonstrated significant regulation of immune-related pathways, such as the Toll-like receptor signaling and phagosome pathways, in gills under ammonia stress [39]. The broad enrichment of “transporter activity” suggests that ammonia stress impacts transmembrane transport processes. This is likely closely associated with the regulation of ion balance and mechanisms for ammonia excretion. These results support the view that multiple organs respond synergistically to ammonia nitrogen stress. Ammonia nitrogen stress triggers a wide range of cell structure reorganization and metabolic reprogramming, in which different tissues assume different stress roles according to their physiological functions.

The DEGs in five tissues of *Megalobrama amblycephala* showed obvious tissue specificity at the level of KEGG annotation and pathway enrichment, but generally still focused on cell growth and death, signal transduction, transport and degradation, immune response, and lipid and amino acid metabolism. It is suggested that ammonia stress not only acts on a single organ, but also disturbs the body’s material metabolism, antioxidant defense, immune homeostasis, and cell fate regulation network. In *Megalobrama amblycephala*, chronic or subchronic ammonia exposure can also cause significant changes in growth, antioxidant and immune indicators [44,45]. Therefore, the synergistic response of multiple tissues observed in this study is consistent with the general law of ammonia toxicity in fish, and it also shows that blunt snout bream is highly sensitive to ammonia stress. The liver itself is a pivotal organ for nitrogen metabolism, detoxification, and energy distribution in fish, and metabolic reprogramming is often the earliest to occur under ammonia exposure. Studies have shown that ammonia exposure can lead to damage to the structure of fish liver tissue and simultaneously change the expression of genes related to glycogen metabolism, the tricarboxylic acid cycle, lipid metabolism, and the urea cycle [19]. Ammonia stress can also significantly affect detoxification metabolism, immune response, and oxidative stress-related processes. Based on this, the enrichment of phagosome, endocytosis, and ferroptosis pathways in this study is more likely to reflect that hepatocytes maintain tissue homeostasis by enhancing the removal of damaged components, inflammation recognition, and oxidative damage response under ammonia stress [46,47]. The gill is the primary interface between the fish body and the external water environment, and it also undertakes the functions of ammonia excretion, ion regulation, and acid–base balance. Therefore, it is usually one of the most sensitive target organs to ammonia toxicity. Studies have shown that acute ammonia exposure can cause gill tissue morphological damage, oxidative stress enhancement, and immune response activation, accompanied by changes in stress defense pathways such as HIF-1α/NF-κB and Nrf2-Keap1 [48,49,50]. The significant enrichment of proteasome and lysosomal pathways in this study indicates that gill tissue may maintain cell homeostasis by accelerating the degradation of damaged proteins and organelles. The enrichment of glutathione metabolism and CYP450-related pathways indicated that gills may have initiated obvious antioxidant and exogenous detoxification mechanisms [51,52]. Ammonia exposure can significantly change the antioxidant enzyme activity, phosphatase level, and inflammation-related gene expression in fish kidney tissues [53,54]. Combined with the results of this study, the co-enrichment of p53, cell cycle, and cellular senescence suggested that DNA damage response, cell cycle arrest, and functional decline may occur in renal cells under ammonia stress [55]. The enrichment of cysteine/methionine metabolism and arginine/proline metabolism indicates that the kidney may buffer ammonia toxicity by regulating both the sulfur-containing antioxidant system and nitrogen-containing metabolic pathway. Although the enrichment of brain tissue was significantly weaker than that of liver, gill, and kidney, its change direction was more focused on “membrane lipid remodeling-barrier stability regulation-protective stress”. Ammonia can cross the barrier of fish brain tissue and cause neurotoxicity, which is characterized by enhanced oxidative stress, brain tissue swelling, and neurological dysfunction. Therefore, the enrichment of the tight junction pathway in this study suggests that ammonia stress may affect the stability of the brain tissue barrier structure [56]. The changes in glycerophospholipid metabolism, unsaturated fatty acid biosynthesis, and FoxO/autophagy-related pathways are more likely to reflect that nerve tissue alleviates ammonia-induced injury through membrane lipid remodeling and cell protection mechanisms [57,58,59,60]. Subchronic ammonia stress can inhibit the expression of muscle growth-related genes in *Megalobrama amblycephala*, accompanied by a decrease in overall growth performance [44,45]. In *Micropterus salmoides*, ammonia exposure can lead to changes in body energy distribution and increased oxidative damage [19]. Combined with the results of this study, the enrichment of oxidative phosphorylation, PPAR, and glycerol metabolism pathways indicated that muscle tissue may meet the energy needs under stress by adjusting mitochondrial energy supply and lipid utilization [61]; the ubiquitin-mediated protein degradation and the activation of the proteasome pathway suggest the elimination of damaged proteins and the acceleration of protein turnover in muscle fibers [62].

## 5. Conclusions

In this study, the RNA-seq method was used to study the transcriptome of five tissues of *Megalobrama amblycephala*. Enrichment analysis indicated that ammonia nitrogen stress may primarily affect immune-inflammatory processes, material transport and degradation, maintenance of protein homeostasis, oxidative stress defense, remodeling of membrane lipid metabolism, and regulation of energy metabolism. In different tissues, genes related to specific functions are enriched according to their physiological roles: in the liver, genes related to detoxification and damage clearance are enriched; in the gill, genes related to barrier defense and antioxidant/detoxification are enriched; in the kidney, genes related to damage response and metabolic regulation are enriched; in the brain, genes related to membrane lipid homeostasis and protective stress are enriched; and in the muscle, genes related to energy metabolism and structural function adjustment are enriched. The results of this study indicate that there may be functional differentiation and synergy among different organs in the stress response. Although there are certain limitations based on transcriptome annotation and enrichment analysis, this work still provides fundamental data for analyzing the mechanism of ammonia nitrogen toxicity, which contributes to a deeper understanding of ammonia nitrogen tolerance and damage in cultured fish.

## Figures and Tables

**Table 1:** Primers used for RT-qPCR verification of DEGs.

DEGs	Forward Primer (5′–3′)	Reverse Primer (5′–3′)	Product Length
FNG	TCATGCGGACTTTACTCGACG	CCATCTTCACTGCATCACGAG	129
SCD	GGCCTAAACCTCCCATCGTC	CGAAACATGCGAAAGTCCAG	135
NAGA	GACAAGGTCCAGATCGACGC	GACATCATTGGGTAGCCTTGC	113
PSMA3	GGCCTTTTGGATGCAGTTTCA	GCACAACCCCAGTAACCGTA	106
YKT6	GCAAGCTGAACTGGACGAAA	TCTGCTTGCGTGCCGTTTTAT	149
NQO1	AATAAGACTCCGCTTCGCAC	GTGCTGTCTTCTGTGCCATTTC	134
HEXB	TATGACGCAGCAGGGCTTTG	TGTGTGTCGGCTTTCAGCTTCA	150
TAT	GCACTATAATCTGCTGCCGGA	ATTGGACGGGTTGTTGACGA	106
ACTC1	AGACTGCTGCTGTGATATTCCC	GTGGTTTAGGCAGGTTTGACAC	130
PIGR	AAGTGACCCTCACAGTCGTC	TTGTCCTTTGATGTTTCGGCT	137
β-actin	TCTGCTATGTGGCTCTTGACTTCG	CCTCTGGGCACCTGAACCTCT [28]	132

**Table 2:** Sample sequencing data quality statistics.

Samples	ReadSum	BaseSum	GC (%)	Q30 (%)
BC1	19,340,230	5,802,069,000	47.92	94.12
BC2	23,283,755	6,985,126,500	46.36	92.9
BC3	23,060,237	6,918,071,100	46.52	93.85
BN1	21,960,036	6,588,010,800	46.2	93.64
BN2	21,364,323	6,409,296,900	45.8	93.45
BN3	21,825,245	6,547,573,500	45.98	93.81
GC1	24,146,564	7,243,969,200	45.89	93.57
GC2	22,847,340	6,854,202,000	45.81	93.27
GC3	26,109,854	7,832,956,200	46.79	93.71
GN1	25,884,683	7,765,404,900	47.21	94.27
GN2	21,410,200	6,423,060,000	46.56	93.66
GN3	25,527,944	7,658,383,200	46.07	93.3
KC1	20,764,607	6,229,382,100	45.89	93.5
KC2	27,650,414	8,295,124,200	46.56	93.76
KC3	25,480,494	7,644,148,200	48.7	93.73
KN1	22,519,809	6,755,942,700	46.92	93.64
KN2	23,980,743	7,194,222,900	46.93	94
KN3	22,456,799	6,737,039,700	46.38	93.5
LC1	23,013,792	6,904,137,600	47.1	94.21
LC2	20,594,531	6,178,359,300	47.4	94.07
LC3	21,988,083	6,596,424,900	47.36	93.77
LN1	22,117,896	6,635,368,800	47.45	94.13
LN2	23,820,658	7,146,197,400	46.73	94.34
LN3	27,040,409	8,112122,700	47.09	93.8
MC1	21,107,270	6,332,181,000	48.67	93.91
MC2	21,191,908	6,357,572,400	48.41	92.85
MC3	21,550,273	6,465,081,900	48.86	93.99
MN1	18,938,920	5,681,676,000	48.84	93.78
MN2	21,255,583	6,376,674,900	49.2	93.35
MN3	19,158,635	5,747,590,500	48.63	93.67

Note: BC, GC, KC, LC and MC represented the control group mixed samples of brain, gill, kidney, liver and muscle, respectively (three parallels in each group). BN, GN, KN, LN and MN represented the experimental group mixed samples of brain, gill, kidney, liver and muscle, respectively (three parallels in each group). A total of 30 samples were used for transcriptome sequencing.

**Table 3:** Sequencing data assembly statistics.

Length Range	Transcripts	Unigenes
300–500	183,720 (21.62%)	95,981 (20.16%)
500–1000	137,901 (16.23%)	69,570 (14.61%)
1000–2000	81,800 (9.63%)	29,856 (6.27%)
2000+	446,291 (52.52%)	280,660 (58.95%)
Total Number	849,712	476,067
Total Length	680,837,014	256,443,725
N50 Length	1812	842
Mean Length	801.26	538.67

**Table 4:** Unigene annotation results statistics.

Anno_Database	Annotated_Number	300 ≤ Length < 1000	Length ≥ 1000
COG_Annotation	18,695	5678	8701
GO_Annotation	39,776	12,818	18,713
KEGG_Annotation	108,414	42,569	31,811
KOG_Annotation	46,341	15,167	19,222
Pfam_Annotation	43,309	12,676	22,435
Swissprot_Annotation	42,110	13,542	19,942
nr_Annotation	110,924	43,561	32,206
All_Annotated	118,320	46,442	32,656

**Table 5:** Statistical table of the number of DEGs.

Type	Total	Up	Down
BC_vs_BN	758	374	384
GC_vs_GN	760	399	361
KC_vs_KN	974	368	606
LC_vs_LN	239	122	117
MC_vs_MN	308	68	240
Total	3039	1331	1708

**Table 6:** Multi-organization partially overlapping DEGs statistics table.

Overlapping Organs	Gene Name	log_2_FoldChange (Former)	log_2_FoldChange (Latter)
B and G	ZNF239	7.84	−7.09
	TC1A	−3.79	−3.57
	DMBT1	6.73	−2.25
G and M	ZNF595	28.05	−13.11
G and L	MHC-I	−11.36	−7.02
	CCL8	−8.32	−7.28
G and K	NLRC3	10.43	8.13
K and L	WFDC5	−30.00	26.63
	H2B.1	−30.00	26.17

Note: Log_2_FoldChange (former) represents the expression level of the gene in the front organ, and log_2_FoldChange (latter) represents the expression level of the gene in the back organ.

**Table 7:** Statistical table of annotated DEGs.

DEG_Set	Total	Swiss-Prot	GO	KEGG	COG	KOG	Pfam	NR
BC_vs_BN	228	71	66	86	26	68	60	213
GC_vs_GN	281	84	81	122	42	84	97	273
KC_vs_KN	430	184	180	178	50	142	170	416
LC_vs_LN	90	49	42	29	14	28	41	88
MC_vs_MN	126	72	70	63	30	58	72	122
Total	1155	460	439	478	162	380	440	1112

## Data Availability

All data supporting this study are available within the paper. For additional raw data files, they can be obtained from the corresponding authors upon reasonable request.

## Abbreviations

The following abbreviations are used in this manuscript:

DEGs	Differentially expressed genes
LC	Liver control group
MC	Muscle control group
BC	Brain control group
GC	Gill control group
KC	Kidney control group
LN	Liver ammonia nitrogen stress group
MN	Muscle ammonia nitrogen stress group
BN	Brain ammonia nitrogen stress group
GN	Gill ammonia nitrogen stress group
KN	Kidney ammonia nitrogen stress group
ZNF239	Zinc finger protein 239
ZNF595	Zinc finger protein 595
TC1A	Transposable element Tc1 transposase
DMBT1	Deleted in malignant brain tumors 1
CCL8	C-C Motif Chemokine Ligand 8
MHC-I	MHC class I antigen
NLRC3	NLR Family CARD Domain Containing 3
H2B.1	Histone H2B.1
WFDC5	WAP four-disulfide core domain 5
